# The Sequence Recall Task and Lexicality of Tone: Exploring Tone “Deafness”

**DOI:** 10.3389/fpsyg.2022.902569

**Published:** 2022-07-12

**Authors:** Carlos Gussenhoven, Yu-An Lu, Sang-Im Lee-Kim, Chunhui Liu, Hamed Rahmani, Tomas Riad, Hatice Zora

**Affiliations:** ^1^Centre for Language Studies, Radboud University, Nijmegen, Netherlands; ^2^Department of Foreign Languages and Literatures, National Yang Ming Chiao Tung University, Hsinchu, Taiwan; ^3^College of Literature and Journalism, Sichuan University, Chengdu, China; ^4^Department of Swedish Language and Multilingualism, Stockholm University, Stockholm, Sweden; ^5^Department of Neurobiology of Language, Max Planck Institute for Psycholinguistics, Nijmegen, Netherlands

**Keywords:** word prosody, lexicon-based memory, tone contrast salience, tone language, semi-tonal language, sequence recall task

## Abstract

Many perception and processing effects of the lexical status of tone have been found in behavioral, psycholinguistic, and neuroscientific research, often pitting varieties of tonal Chinese against non-tonal Germanic languages. While the linguistic and cognitive evidence for lexical tone is therefore beyond dispute, the word prosodic systems of many languages continue to escape the categorizations of typologists. One controversy concerns the existence of a typological class of “pitch accent languages,” another the underlying phonological nature of surface tone contrasts, which in some cases have been claimed to be metrical rather than tonal. We address the question whether the Sequence Recall Task (SRT), which has been shown to discriminate between languages with and without word stress, can distinguish languages with and without lexical tone. Using participants from non-tonal Indonesian, semi-tonal Swedish, and two varieties of tonal Mandarin, we ran SRTs with monosyllabic tonal contrasts to test the hypothesis that high performance in a tonal SRT indicates the lexical status of tone. An additional question concerned the extent to which accuracy scores depended on phonological and phonetic properties of a language’s tone system, like its complexity, the existence of an experimental contrast in a language’s phonology, and the phonetic salience of a contrast. The results suggest that a tonal SRT is not likely to discriminate between tonal and non-tonal languages within a typologically varied group, because of the effects of specific properties of their tone systems. Future research should therefore address the first hypothesis with participants from otherwise similar tonal and non-tonal varieties of the same language, where results from a tonal SRT may make a useful contribution to the typological debate on word prosody.

## Introduction

Lexical tone has been investigated in a large body of perception research and is a prominent traditional typological concept in phonology, perhaps more so than word stress, which until recently was often treated as a universal (*cf*. van Heuven and Turk, 2020). Tones can form a great variety of subsystems in the phonologies of languages. There can be few or many of them and contrasts will vary in salience. Functionally, they could share the phonological specification of morphemes with vowels and consonants (“lexical tones”) or be their sole exponents (“grammatical tones,” Hyman, 2011, 2016). While the linguistic and cognitive evidence for lexical tone is beyond dispute, as indicated by the results of dichotic listening, categorical perception, ABX designs, and brain response registrations (Lau et al., 2020), the word prosodic systems of many languages continue to escape the categorizations of typologists, with frequent debates about the categorization of tone languages (Hyman, 2006; Kehrein et al., 2017; Steien and Yakpo, 2020; Gooden, 2022). The present paper aims to contribute to the understanding of the lexical status of tone by comparing non-tonal, semi-tonal, and tonal languages in a Sequence Recall Task (SRT). It was developed by Emmanuel Dupoux and colleagues as a diagnostic for the presence of word stress in a language (Dupoux et al., 2001). It followed their earlier speculations on why French listeners underperformed in an ABX task relative to Spanish listeners, where A and B were trisyllabic non-words differing in the location of stress (Dupoux et al., 1997). An SRT trial presents participants with a sequence of some 4 to 6 disyllabic non-words which have a prominence on either one or another of its syllables, as in the disyllabic non-word sequence *númi – numí – númi – númi*. Participants are asked to reproduce the order of the two non-words on a keyboard (in this case 1–2–1–1) after hearing a distracting sound immediately after the sequence, intended to prevent them from relying on their acoustic memory (cf. Baddeley, 2010). Speakers of Spanish, a language with contrastive word stress, outperformed speakers of French on this task, which language has phrasal stress (Dupoux et al., 2001). The effect survives language contact as in L2 learning (Dupoux et al., 2008).

Explanations of the inability of French listeners to perform the task as effectively as Spanish listeners first addressed the exposure to meaningful word prosody during language acquisition, but later shifted to the resulting abstract lexical representation of stress (Peperkamp, 2004; Dupoux et al., 2008). Providing support for this interpretation, Rahmani et al. (2015) showed that the presence of syllabic prominence in lexical representations, whether from tone or stress, explained the results of an experiment with five language groups, Dutch, Japanese, French, Indonesian, and Persian. As hypothesized, Dutch and Japanese participants outperformed the participants in the other three language groups, who for that reason are “stress-deaf” (the term is due to Dupoux et al., 1997). The explanation the authors give is that Dutch and Japanese participants could engage their lexicon-based memory on the basis of the contrastive location of a syllabic prominence in words, stress in Dutch and a HL melody in Japanese. The interpretation of stress as tone by the Japanese listeners was also evident in Qin et al. (2017), in which Standard Mandarin, Taiwan Mandarin, and English participants achieved comparable SRT performance on disyllabic English stress pairs. None of the other three languages in Rahmani et al. (2015) possesses lexically contrastive word prosody, whether due to stress or tone, so that any reliance on a “lexical memory” is not an option open to them.

The similar effects of stress and tone in the Dutch and Japanese accuracy scores in Rahmani et al. (2015) must not lead us to lose sight of the profoundly different character of tone from stress. Tones can form a great variety of subsystems in the phonologies of languages. There can be few or many of them and contrasts will vary in salience. And they could be lexical as well as morphological or syntactic (‘grammatical’). Stress, by contrast, is usually taken to be the head of a constituent of the prosodic hierarchy, the foot, in which unstressed syllables may additionally occur in non-head positions (Selkirk, 1980; Hayes, 1995). Since all words are footed, and hence stressed, no stress contrasts are possible on monosyllables if a language has feet (“obligatoriness,” Hyman, 2006). This is why the non-words in a stress-based SRT are disyllabic: stressed–unstressed or unstressed–stressed. At the same time, this makes it necessary to use monosyllabic contrasts in the case of tone, in order to guarantee tonal interpretations of the pitch contrasts. It is true that stress systems too vary across languages, for instance in the degree of exceptionality of stress locations. Moreover, stressed syllables may or may not have an intonational pitch accent, as in Germanic languages (*cf*. “primary stress,” Domahs et al., 2008), and stress may correlate with syllable quantity or vowel reduction (Hayes, 1995). Such differences have not affected the results of SRTs much. In Peperkamp and Dupoux (2002), an experiment with six language groups, Polish, which has regular penultimate stress with few words having ultimate or antepenultimate stress, came out as intermediate between a stress-deaf and a non-stress-deaf group. Also, the categorical interaction between vowel quality and stress in European Portuguese explains why listeners are stress-deaf if they cannot rely on the vowel quality differences (Correia et al., 2015; Lu et al., 2018).

Because of the more varied complexity of lexical tone systems compared to stress systems, we may reasonably expect the results of a tonal SRT to be affected by relevant features of a language’s phonology (Best, 2019). First, the number of monosyllabic tone melodies may vary from 2 to as many as 9 (e.g., Hyman, 2011). A high functional load of lexical pitch contrasts may well affect recall accuracy. Moreover, tone contrasts may be restricted to certain positions in the word, like the final syllable in Ma’ya (Remijsen, 2002) or a non-final syllable in Swedish (Riad, 2014: 182). This means that in addition to a simple discrete concept of lexical “tonality,” that is, the presence of a pitch specification in the phonological form of at least some morphemes (Hyman, 2006), it will be necessary to test for effects of relative “tonality,” that is, the complexity of lexical tone systems. Second, the choice of the pitch contrast in the experiment may favor participants that happen to have that contrast in their tonal grammar. We take this potential benefit to be independent of the lexical or intonational status of the pitch contrast. An experiment that intends to include this factor in its design, will need to test for a number of pitch contrasts, such that each of them fails to turn up in at least one language under investigation. Third, pitch contrasts vary in salience, that is, in the perceptual difference between the two contrasting pitch shapes. If sequences of less salient contrasts are harder to recall than contrasts with larger differences, the size of the contrast will need to be included as a variable in our experiment.

We selected one unambiguously non-tonal language (Indonesian), one borderline case (Stockholm Swedish), and two unambiguously tonal languages (Taiwan Mandarin and Zhumadian Mandarin). The inclusion of two similar tone languages served as a sanity check, as it predicts that their scores will be quite similar as well as quite different from the non-tonal language. A heuristic element in our choice of languages is the ambiguous “semi-tonal” language, which might statistically side with either the non-tonal language or the tonal ones, or appear as a category in between.

**Figure 1:**
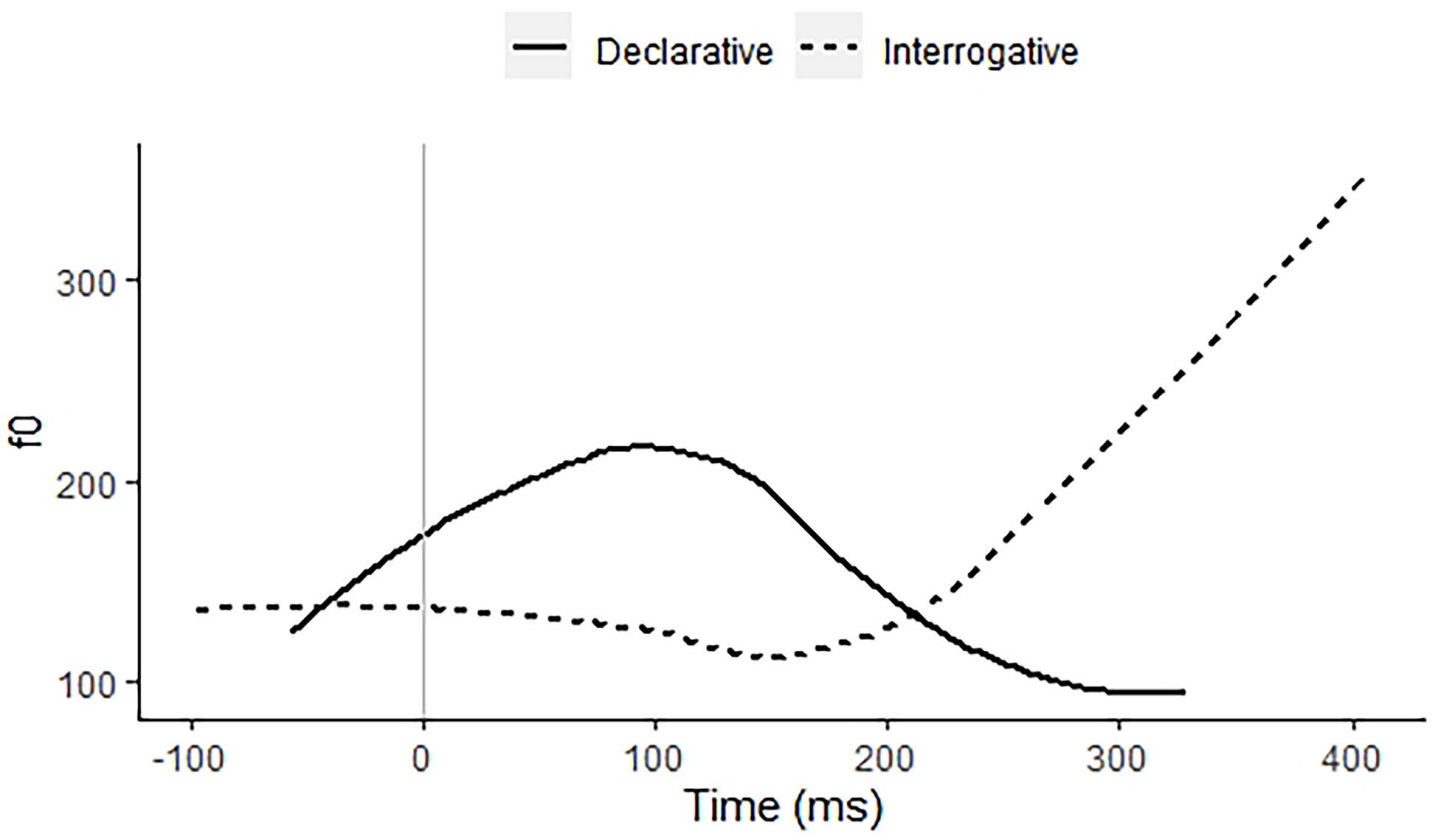
f0 contours of declarative (solid line) and interrogative (dashed line) citation pronunciations of the monosyllabic word *gong* (“gong”), recorded by a 28-year-old male speaker of Standard Indonesian.

*Indonesian* has neither tone nor stress on any syllable, whether word-based or phrase-based (Odé, 1994; Goedemans and van Zanten, 2007; Maskikit-Essed and Gussenhoven, 2016). The performance of the Indonesian participant group should provide a lower baseline. The language has an intonational contrast between a phrase-final rise, used in pre-final intonational phrases and in final interrogative phrases, and a rise–fall, used in final declarative phrases. The contrast between these right-edge melodies will show up in stated and questioned monosyllabic words. Figure 1 shows this contrast as spoken by a 28-year-old male speaker from East Java. This pitch contrast is the main intonational contrast in the language and there may therefore be a fair bit of variation in the phonetic shapes.

**Figure 2:**
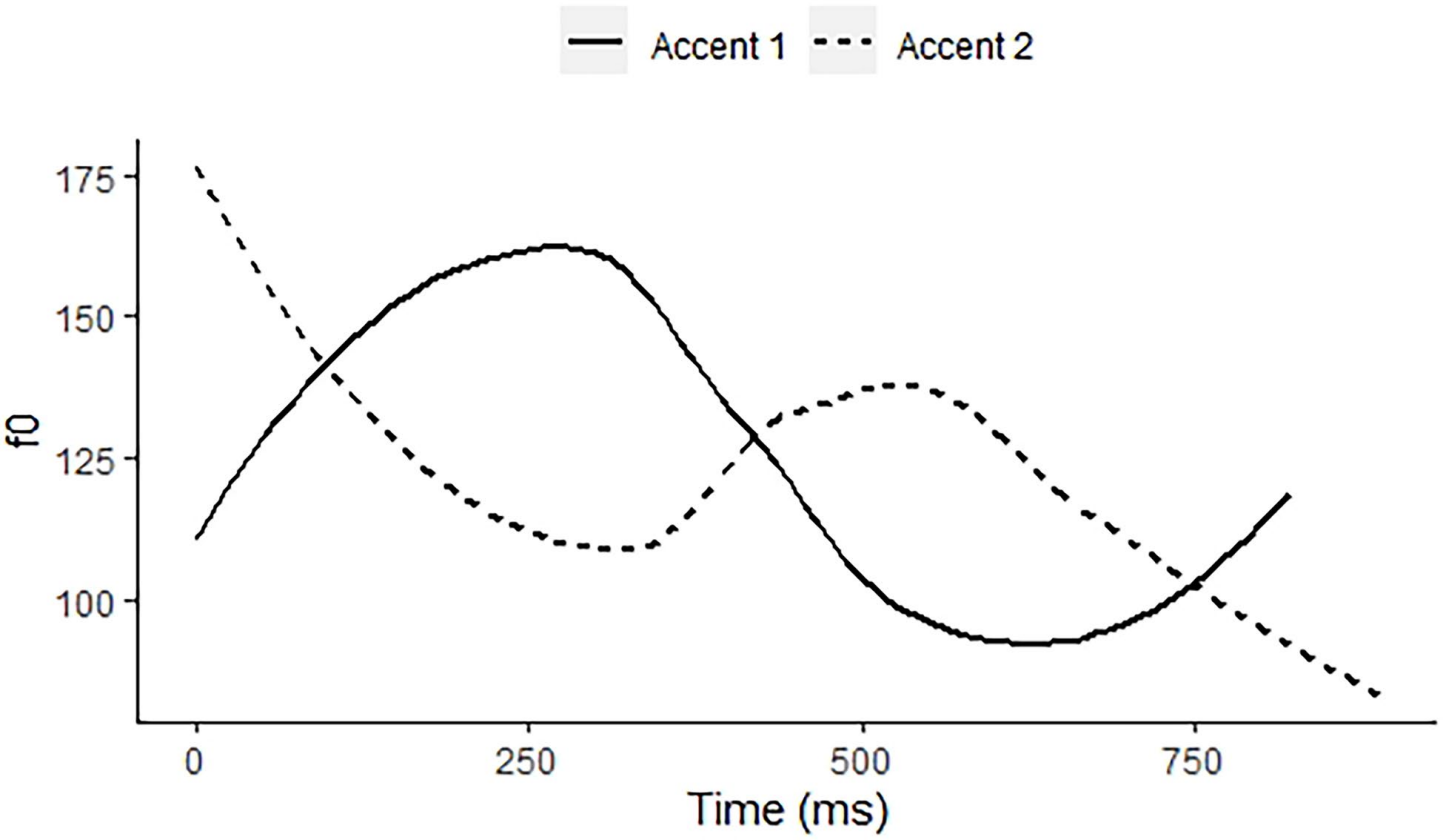
f0 contours of citation pronunciations of Accent 1 on anden “the duck” (solid line) and Accent 2 on anden “the spirit” (dashed line) by a 60-year-old male speaker of Stockholm Swedish.

*Stockholm Swedish* has a lexical tone contrast in non-final syllables with word stress, Accent 1 vs. Accent 2, as occurring in *anden* “the duck” and *anden* “the spirit,” respectively. Accent 1 is a rise in the stressed syllable, followed by low pitch when occurring in the nuclear position, as illustrated by the solid line of an isolated pronunciation of the expression meaning “the duck” in Figure 2. Accent 2 has an early fall in the stressed syllable, which in the nuclear position is followed by a pitch peak in the phrase-final syllable, as shown by the dashed line for an isolated pronunciation of the expression meaning “the spirit” in Figure 2. Both have an intonational melody LHL%, which is preceded by a lexical H in the case of Accent 2, effectively shifting the intonational f0 peak onto the final syllable (Riad, 2014). Arguably, the different contours in the unstressed phrase-final syllables represent contrasting phonetic cues to the tone contrast on the penultimate syllable. However, such contextual cues abound in languages generally, so that we cannot interpret the phrase-final pitch difference as a contrast of the language, whether lexical or intonational.

**Figure 3:**
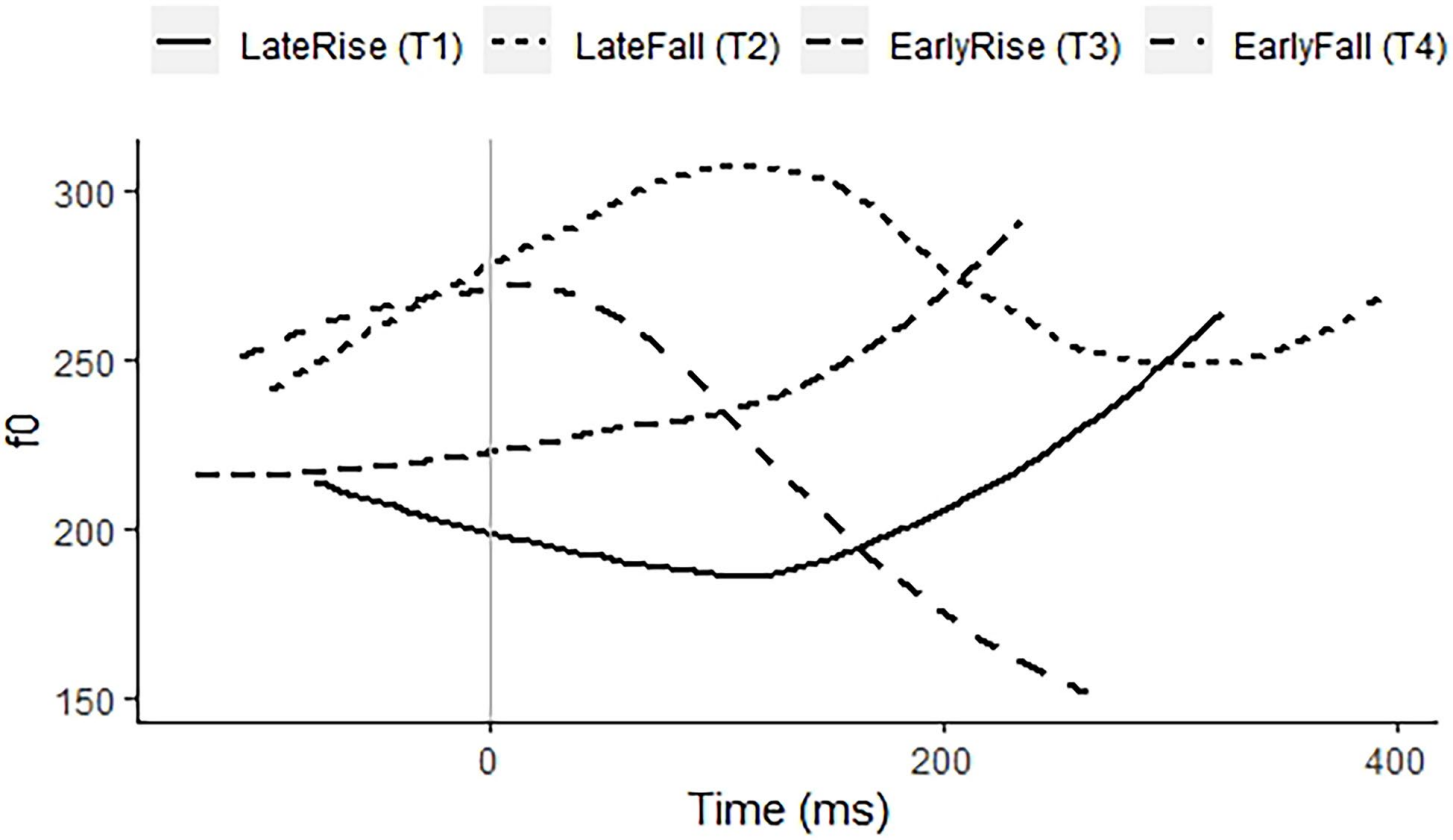
f0 contours of citation pronunciations of a late rise/Tone 1 on 麥 “cereal,” a late fall/Tone 2 on 埋 “bury,” an early rise/Tone 3 on 買 “buy,” and an early fall/Tone 4 on 賣 “sell,” all with the segmental syllable /mae/, recorded by a 22-year-old female speaker of Zhumadian Mandarin.

*Zhumadian Mandarin*, spoken in Henan Province, China, has four lexical tones, two rises, and two falls, which contrast for temporal alignment, leading to a late rise (Tone 1), a late fall (Tone 2), an early rise (Tone 3), and an early fall (Tone 4). The early rising Tone 3 tends to rise only a little, thus resembling Tone 1 of Standard Mandarin, while the late rising Tone 1 may sound like a final, dipping Tone 3 of Standard Mandarin (Gussenhoven and van de Ven, 2020). The language has a Fourth Tone Sandhi rule, changing 4 + 4 into 1 + 4, as well as toneless morphemes, that is, neutral tone. Figure 3 presents examples of the four tones on the syllable /mae/. Younger speakers are bilingual with Standard Mandarin. Except in educational contexts, speakers use the Zhumadian dialect.

**Figure 4:**
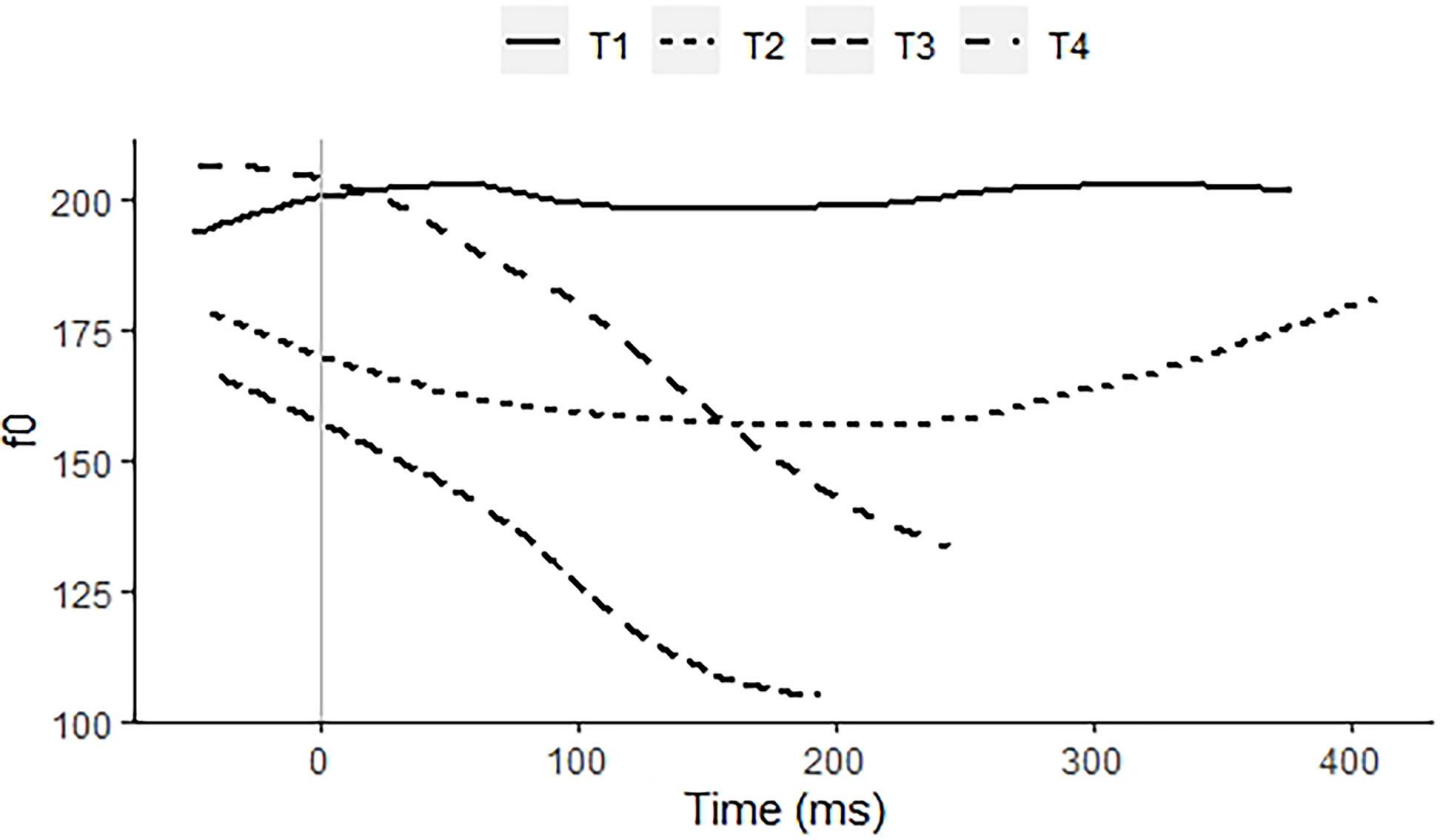
f0 contours of citation pronunciations of a high level/Tone 1 on 媽 “mother,” a rise/Tone 2 on 麻 “hemp,” a low tone/Tone 3 on 馬 “horse,” and a high falling/Tone 4 on 罵 “scold,” all with the segmental syllable/ma/, recorded by a 40-year-old female speaker of Taiwan Mandarin.

*Taiwan Mandarin* is a standard variety of Mandarin. It has four lexical tones, a high level tone, a rising tone, a low tone, and a high falling tone, Tones 1 to 4, respectively (Figure 4). In addition, it has the Third Tone Sandhi rule (3 + 3 → 2 + 3) as well as syllables with neutral tone, whose pitch contours are derivative from a preceding toned syllable. The most striking difference with Standard Chinese is the shorter duration of Tone 3, which typically lacks or significantly reduces the rising part in phrase-final position (Kubler, 1985; Fon and Chiang, 1999; Torgerson, 2005; Deng et al., 2006). Its tonal complexity is quite comparable to that of Zhumadian Mandarin.

## Materials and Methods

**Figure 5:**
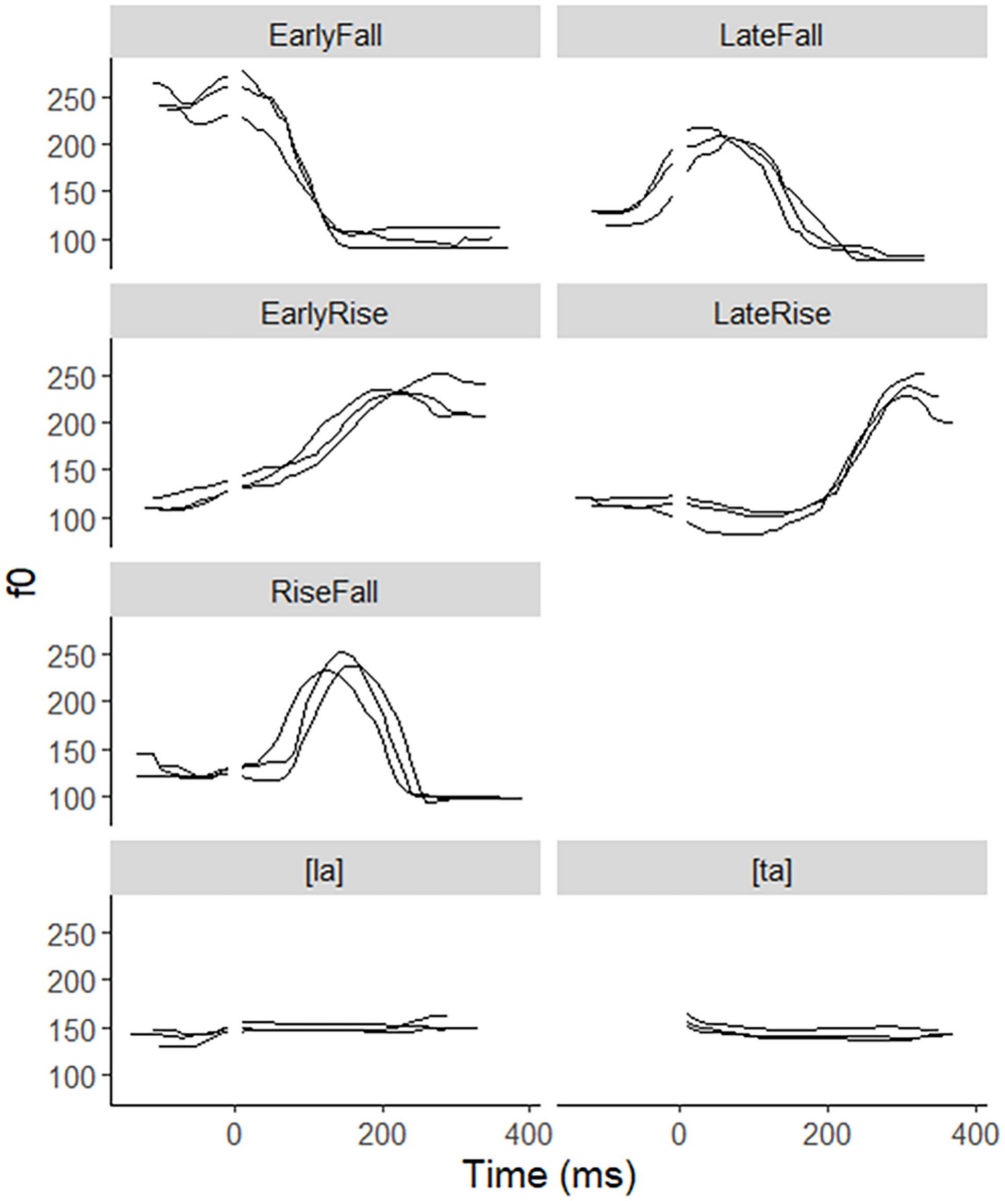
f0 tracks of the three tokens for each of 7 syllables types, with the onset-vowel boundaries indicated by an interruption.

**Figure 6:**
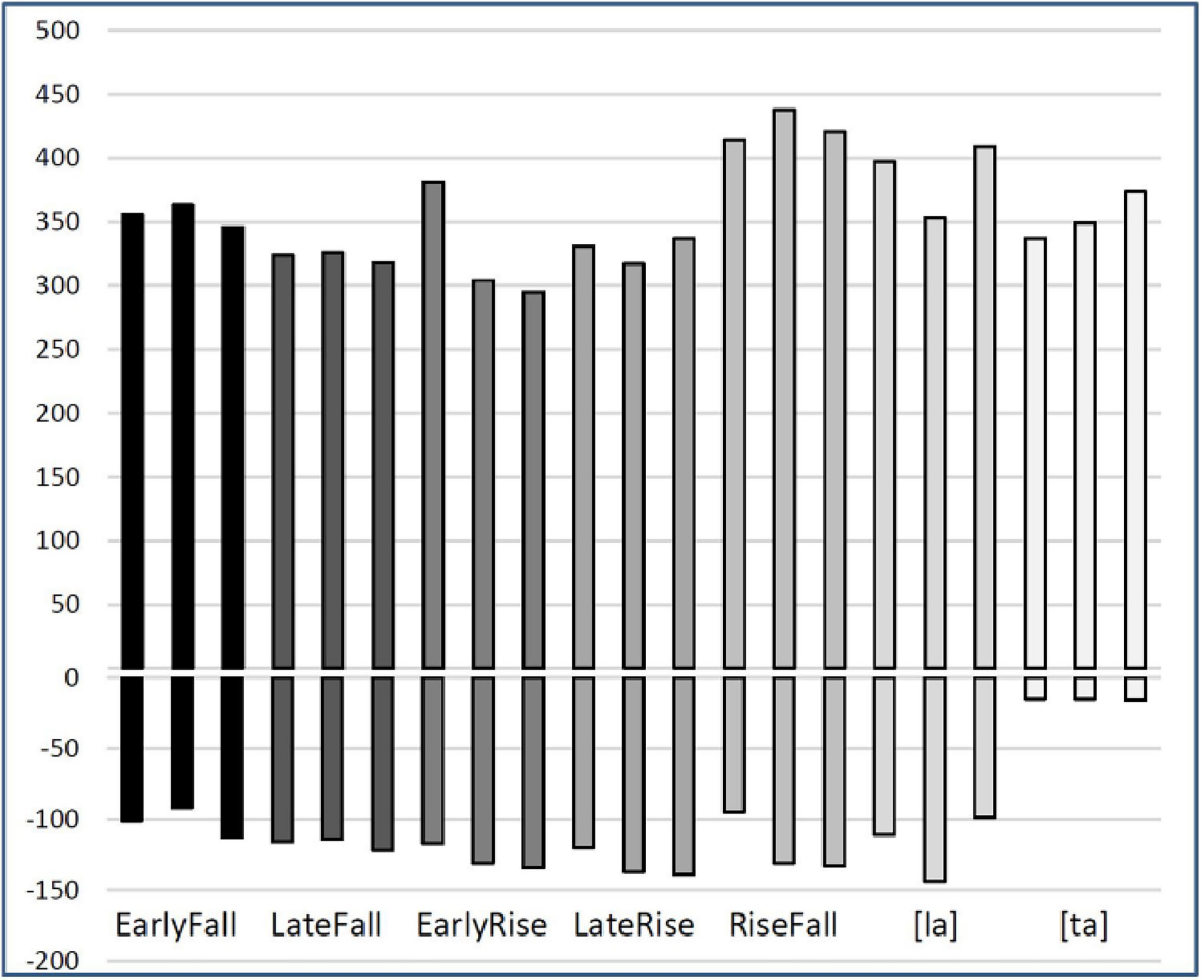
Durations of onset [l] (negative bars) and rhyme [a] (positive bars) of the 27 stimuli in the experiment. For [ta], the negative bars give the positive VOTs. The value for the onset in [ta] is the burst and friction of the released [t].

We included three-pitch contrasts in the experiment, EarlyFall vs. LateFall, EarlyRise vs. LateFall, and RiseFall vs. EarlyRise. None of these are pitch levels, which are likely to sound like a melody when occurring in a sequence, which would be more memorable than sequences of pitch shapes. In addition, we used a “phoneme” contrast of the type that has served as a control variable in SRT experiments (Peperkamp et al., 2010; Rahmani et al., 2015; Qin et al., 2017). A phonetically trained speaker of Dutch in his early 70s recorded each of these seven syllable types at least eight times in a sound-treated booth. Three tokens of each syllable type were selected that sounded natural and seemed good exemplars of the intended pitch shape. Figure 5 displays these tokens for all five-pitch shapes figuring in these contrasts, all pronounced on the syllable [la], aligned at the onset-vowel boundary indicated by the gap in the figure, which corresponds to 0 ms in the signal. The phoneme contrast was between the syllables [ta] and [la], both pronounced with level midpitch. We avoided adjustments of the original durations, unlike Peperkamp et al. (2010), who drastically shortened the original recordings of disyllables. Largely depending on pitch shape, tones require a certain duration to produce (Xu and Sun, 2002) and shortened syllables may as a result sound distorted. Across pitch shape types, durations varied from 430 ms for a token of the EarlyRise to 569 ms for a token of the RiseFall. The three tokens had very similar durations in three of the five-pitch shape types. Only the triplets for the EarlyFall and the LateFall varied more noticeably, for which reason we standardized the three exemplars to the rounded mean duration in each triplet, 440 ms and 460 ms, respectively, using Praat (Boersma and Weenink, 1992–2020). Figure 6 shows acoustic durations of all 15 pitch shape stimuli and the 6 stimuli for the phoneme contrast, for onset consonant and vowel separately; in the case of [ta], the burst duration is shown.

A number of independent variables were included in the analysis. Sex and aptitude were the two participant variables, of which aptitude was motivated by the expectation that participants may vary in their aptitude for carrying out an SRT. For this variable we used each participant’s mean accuracy score on the phoneme contrast. Rather than controlling for pitch discrimination and categorization abilities, which have been shown to explain variation in pitch-related learning and identification tasks (*cf*. Sadakata and McQueen, 2014; Zhao and Kuhl, 2015; Bowles et al., 2016; Qin et al., 2021; Rhee et al., 2021), we intended to control for a more general ability to perform the experimental task of remembering sequences of tokens of two sound categories. Earlier research had taken this effect for granted, by subtracting phoneme accuracy scores from stress contrast scores (e.g., Peperkamp et al., 2010). We felt we needed to have a better understanding of the relation between the control and experimental contrasts in view of the prospect of continued research on languages with older populations of speakers.

Four language variables figured in our investigation, lexicality, tonecomplexity, salience, and havecontrast. Since our main hypothesis was that participants with tonal language backgrounds will outperform participants with non-tonal language backgrounds, we interpret lexicality as a binary variable characterizing any language with a lexical marking of pitch as tonal (Hyman, 2006), which includes “semi-tonal” Swedish. While the distribution of the two Swedish tone categories is highly predictable from the phonology and morphology of words (Bruce, 1977: 18; Wetterlin et al., 2007; Riad, 2014: 183), there are exceptions, most obviously in disyllables with penultimat stress. For instance, many loan words have Accent 1, like *ketchup* and *solo*, in contrast to other words, like *senap* “mustard” and *pizza*, which have Accent 2. Moreover, in a priming experiment, Althaus et al. (2021) have shown that native speakers use the contrast in lexical access. Accordingly, only Indonesian was coded as −1 and the other three as 1 for this variable. At the same time, a gradient characterization of lexical tone complexity might provide a better predictor of accuracy scores than binary lexicality, for which reason we coded the two Mandarin varieties as 4.0 for tonecomplexity, to reflect the number of tone categories. While Swedish has two tone categories, it has no tone contrast on monosyllabic words and hence not for the monosyllabic non-words in our experiment. We coded it as 0.5, while Indonesian was coded 0.0. Because lexicality and tonecomplexity amount to discrete and gradual interpretations of a language’s status as a tone language, we will not include both variables in the same analysis.

Our experiment involved pitch contrasts that obviously varied in salience. Because sequences of similar pitch shapes may be harder to recall than sequences of more different pitch shapes, we measured subjective phonetic differences among six-pitch shapes, one token of each of the five-pitch shapes in our experiment plus a FallRise, spoken by the same speaker, for the sake of symmetry in the set of pitch shapes to be measured. The 6 × 5 pairs were included in a Praat Multiple-Forced Choice experiment together with two filler pairs, presented in a per participant randomized order. Eight phonetically trained judges were asked to rate all pairs for phonetic distance on a 10-point scale, after listening to recordings of all six-pitch shapes and rating three trial pairs. Pearson’s correlation coefficients between the scores of each judge and the mean score over all judges showed that the scores by two judges failed to reach significance at a 5% level. Of the other six, two judges had *r* < 0.55 and four *r* > 0.83.^1^ They were native speakers of Dutch, English, Korean, and Mandarin (3), with ages ranging from 27 to 47. The native speaker of Korean grew up speaking tonal Gyeongsang Korean, but uses Standard Korean in virtually all domains. Their language backgrounds were otherwise evenly divided over tonal and non-tonal languages, which minimized language-specific biases (*cf*. Huang and Johnson, 2010). Table 1 presents all scores, pooled over the two orders in each pair, which we used as the salience scores.

**Table 1:** Mean subjective phonetic distances per pair of pitch shapes.

	EarlyFall	LateFall	EarlyRise	LateRise	RiseFall	FallRise
LateFall	**2.3**					
EarlyRise	9.5	**8.3**				
LateRise	9.5	8.7	4.3			
RiseFall	7.5	6.8	8.3	**8.7**		
FallRise	8.6	8.0	8.8	8.3	7.2	

Experimental contrasts are printed in bold.

Finally, in order to be able to assess the extent to which the presence of a contrast in the participant’s native language influences accuracy scores, we coded languages for havecontrast for each pitch contrast. When a language has a contrast in its lexical or postlexical phonology, it is coded 1 for that contrast, otherwise −1. For instance, Zhumadian Mandarin has a lexical contrast between an early aligning and a late-aligning fall, while the other three languages do not, entitling it to a 1 coding for that contrast (see Table 2). It also has a contrast between a late fall and an early rise, corresponding to our second experimental contrast. Taiwan Mandarin has a contrast between a fall and a late rise. Native speaker reactions suggest that the EarlyFall and the LateFall are equally good exemplars of the Taiwan Mandarin Fall, while the EarlyRise and the LateRise are both good exemplars of the Taiwan Mandarin Rise. We therefore also coded both Zhumadian and Taiwan Mandarin as 1 for the LateFall vs. EarlyRise contrast. Indonesian has an intonational contrast between a LateRise and a RiseFall, while the other three languages do not. Swedish lacks monosyllabic contrasts, so that it is harder to define the occurrence of our experimental contrast in the phonology of Swedish. Even if we were to interpret the f0 shapes of the first syllables as a RiseFall for Accent 1 and an EarlyFall for Accent 2 (see Figure 2), this would not correspond to any of the experimental pitch contrasts. Accordingly, all three contrasts are coded as −1 for Swedish.

**Table 2:** Experimental pitch contrasts functioning as phonological contrasts.

Contrast	Indonesian	Swedish	Zhumadian Mandarin	Taiwan Mandarin
EarlyFall vs. LateFall	–1	–1	1	–1
LateFall vs. EarlyRise	–1	–1	1	1
LateRise vs. RiseFall	1	–1	–1	–1

We employed two sequence lengths for the two non-words, a 4-non-word and a 5-non-word sequence length, giving a binary variable sequencelength. Piloting with 6-non-word sequences made it clear that these were too difficult to deal with. In addition, we found that the task required a high level of concentration, which we felt put strict limits on the time participants could be asked to perform it. In a further attempt to make the task easier, we blocked the 4-non-word and presented these before moving on the block of 5-non-word sequences. Finally, group and contrast were the variables of central interest in the investigation. A summary of the independent variables introduced above appears in Table 3. Sequences of non-words avoided regular alternations (e.g., 1,212) and maximized the number of switch points (1 to 2, 2 to 1), following Rahmani et al. (2015), which led us to use 1211, 1221, 2112, 2122, 2212 and 1121 for 4-word sequences and 11221, 12112, 12212, 22112, 21221 and 21121 for 5-word sequences. With four contrasts and twice six sequences the total number of trials was 48. The total duration of the experiment was about 30 min.

**Table 3:** Independent variables in the investigation.

Variable		Description
Experimental design	group	Indonesian, Swedish, Zhumadian Mandarin, Taiwan Mandarin
	contrast	EarlyFall vs. LateFall, LateFall vs. EarlyRise, LateRise vs. RiseFall, [la] vs. [ta]
	sequencelength	4-word sequence—1, 5-word sequence 1
Participant	sex	Female—1, Male 1
	aptitude	Accuracy score [la]-[ta]
Linguistic structure	lexicality	Indonesian—1, all other groups 1
	tonecomplexity	Indonesian 0.0, Swedish 0.5, Zhumadian Mandarin 4.0, Taiwan Mandarin 4.0
	havecontrast	See detailed coding in Table II.
	salience	EarlyFall vs. LateFall 2.3, LateFall vs. EarlyRise 8.3, LateRise vs. RiseFall 8.7

We recruited minimally 20 participants for each language who were between 18 and 30 years old and attended or had attended institutes of tertiary education. Table 4 lists the numbers per language split over the sexes, their age ranges, mean ages, and recruitment locations. We presented the experiment on a desktop computer with E-Prime 3.0 for the Zhumadian Mandarin participants and E-Prime 2.0 for the other participants (Schneider et al., 2012). Participants listened individually to the stimuli through headphones. Instructions were provided in English on the screen, supplemented with oral instructions in each native language. The experiment consisted of four blocks, one for each of the four contrasts with breaks in between, in a randomized order for each participant. Each block started with a training session. For the phoneme contrast, participants were trained to associate the syllable [la] with key “1” and [ta] with key “2,” while for the three-pitch contrasts they were trained to associate [LateFall] with key “1” and [EarlyFall] with key “2,” [LateFall] with key “1” and [EarlyRise] with key “2,” and [LateRise] with key “1” and [RiseFall] with key “2.”

**Table 4:** Participants in four language groups.

	N	Age range	Mean age	Location
Indonesian	10F, 10 M	19–30	24.4	National Yang Ming Chiao Tung University (Hsinchu, Taiwan)
Swedish	11F, 10 M	20–29	24.1	Stockholm University (Sweden)
Zhumadian M	15F, 10 M	18–23	19.8	Huanghuai College (Zhumadian, China)
Taiwan M	10F, 10 M	20–22	21.5	National Yang Ming Chiao Tung University (Hsinchu, Taiwan)

Participants were told at the beginning of each block that they were going to learn two words in a foreign language. First, they heard all three tokens of one non-word with a “1” displayed on the screen, and then heard all three tokens of the other non-word with a “2” displayed on the screen. This cycle was repeated three times, exposing participants to 3 tokens x 2 non-words x 3 repetitions, or 18 non-words, before they proceeded to the second training stage, during which they heard each of the 6 tokens, together with a display of the corresponding key on the screen, in a random order. After they had indicated having learned the relevant two-way classification, participants moved on to an identification task in which they heard one of the six tokens in a contrast and were asked to respond by pressing “1” or “2.” After each identification trial, they saw either “CORRECT!” or “INCORRECT!” on their screen for 800 ms as feedback. This procedure was repeated four times. The SRT proper was preceded by a warm-up block with six 3-word sequence trials. No feedback of any kind was given in the 4-sequence and 5-sequence experimental blocks. Ignoring the warm-up block, the experimental trials presented participants with all 48 stimulus pairs (6 sequences × 2 sequence lengths × 4 contrasts). Participants confirmed the completion of their response by pressing the ENTER key. The order of presentation of all sequences within all blocks was randomized per participant. Within each sequence, the non-words were randomly instantiated by one of the three tokens, while no token appeared more than once in a sequence.

Tokens were separated by 120-ms intervals in all sequences. Participants could only register their response after hearing a 1,600-ms recording of four piano chords, played 100 ms after the last token in a sequence. Its function was to reduce the ability of participants to rely on their acoustic memory, similar to that of the recording of “OK!” which has been used for SRTs with stress contrasts. Intervals between trials were 1,500 ms. No response was registered if its sequence length did not match that of the input sequence length.

## Results

Two analytical procedures were followed, after Peperkamp et al. (2010), one to answer the question what properties of the pitch contrast, the languages and the participants predict the accuracy scores and another to establish the differences between language groups and any interactions with the contrasts. Thus, we first report two multiple logistic regression analyses of the linguistic variables salience and havecontrast, together with the participant variables sex and aptitude. In the first multiple logistic regression analysis, we included the binary variable lexicality, while the gradient variable tonecomplexity was included in the second. We will next move on to building a mixed-effects model with the experimental design variables, including the phoneme control contrast [la] vs. [ta] (aptitude).

The results of the multiple logistic regression analysis on the accuracy scores for the three-pitch contrasts with salience, havecontrast, sex, aptitude, and the binary variable lexicality are given in Table 5. Significant havecontrast (*β* = 0.29, *p* < 0.0001) shows that participants generally have higher accuracy scores if some pitch difference they are judging is contrastive in their native language (“yes” *M* = 0.63 vs. “no” *M* = 0.49). salience (*β* = 0.29, *p* < 0.0001) indicates that the participants’ performance relied to a large extent on how salient a specific contrast is. lexicality (*β* = 0.3, *p* < 0.0001) also explained the accuracy results. Participants who speak a (semi-)tonal language (*M* = 0.58) outperformed Indonesian participants, whose native language lacks lexical tone (*M* = 0.39). Lastly, participants’ performance on the three-pitch contrasts strongly depended on their scores for the phoneme contrast (aptitude, *β* = 1.12, *p* < 0.0001). The near-significant effect of sex (*β* = −0.07, *p* = 0.079) weakly indicates that women (*M* = 0.55) performed better than men (*M* = 0.52). The model fit (*r*^2^) is 0.24.

**Table 5:** Results of a multiple logistic regression analysis with tone complexity as the tonality variable.

	*R*^2^ = 0.24				Accuracy means
	B	SE	*z*	*p*	
*Intercept*	−2.709	0.235	−11.515	<0.0001	
havecontrast	0.288	0.042	6.809	<0.0001	no: 0.49; yes: 0.63
salience	0.290	0.014	20.533	<0.0001	2.3: 0.27; 8.3: 0.67; 8.7: 0.67
aptitude	1.123	0.286	3.927	<0.0001	
lexicality	0.302	0.066	4.61	<0.0001	−1: 0.39; 1: 0.58
sex	−0.071	0.04	−1.751	0.079	female: 0.55; male: 0.52

The results of the multiple logistic regression analysis with gradient tonecomplexity instead of lexicality are given in Table 6. With a model fit (*r*^2^) of 0.25, the explained variance is comparable, while the overall results for all identical variables are the same in the two analyses. The range of the accuracy means for tonecomplexity (0.39 to 0.63) is marginally wider than that for lexicality (0.39 to 0.58) in the first analysis.

**Table 6:** Results of a multiple logistic regression analysis with tone complexity as the tonality variable.

	*R*^2^ = 0.25				
	B	SE	*z*	*p*	Accuracy means
*Intercept*	−3.17	0.207	−15.289	<0.0001	
havecontrast	0.181	0.046	3.954	<0.0001	
salience	0.296	0.014	20.668	<0.0001	
aptitude	1.374	0.233	5.905	<0.0001	
tonecomplexity	0.165	0.025	6.533	<0.0001	0.0: 0.39; 0.5: 0.49; 4.0: 0.63
sex	−0.07	0.04	−1.74	0.081	

**Figure 7:**
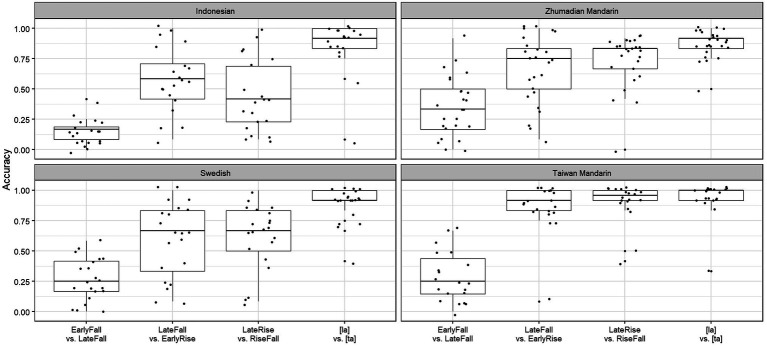
Boxplots and scatterplots for four contrasts and four language groups.

Next, two mixed-effects logistic regression analyses were performed on the accuracy scores to establish the effects of contrasts and language groups. The first focused on the tonally intermediate Swedish. With the Swedish participants and the phoneme contrast, [la] vs. [ta], as baselines, the regression model was fitted with contrast * group and sequencelength as variables, where contrast has the three-pitch contrasts and the phoneme contrast as levels. In addition, the model included random intercepts for participant as well as by-participant random slopes for contrast and sequencelengh. The second analysis was carried out to assess the degree of similarity between the two tonal languages, Taiwan vs. Zhumadian Mandarin. For this analysis, Taiwan Mandarin and the phoneme contrast were set as baselines, with the rest of the model structure remaining the same as that of the first. The analyses were run in R using the *lme4* package (Bates et al., 2015). The results of the two analyses are presented in Tables 7 and 8. Figure 7 gives a box plot with accuracy means and per participant scatter plots.

**Table 7:** Results of mixed-effects logistic regression analysis with Swedish and [la] vs. [ta] as baselines.

	*R*^2^ = 0.47				
	B	SE	*z*	*p*
*Intercept*	2.318	0.300	7.716	<0.0001
*GroupIndonesian*	0.025	0.428	0.059	0.953
*GroupZhumadian M.*	−0.187	0.274	−0.681	0.496
***GroupTaiwan M.***	**0.615**	**0.327**	**1.882**	**0.060**
***ContrastEarlyFall* vs. *LateFall***	**−3.510**	**0.321**	**−10.925**	**<0.0001**
***ContrastLateFall* vs. *EarlyRise***	**−1.794**	**0.309**	**−5.802**	**<0.0001**
***ContrastLateRise* vs. *RiseFall***	**−1.703**	**0.354**	**−4.819**	**<0.0001**
***Sequence***	**−0.421**	**0.044**	**−9.664**	**<0.0001**
*GroupIndonesian:ContrastEarlyFall* vs. *LateFall*	−0.718	0.473	−1.520	0.129
***GroupZhumadian M.:ContrastEarlyFall* vs. *LateFall***	**0.738**	**0.337**	**2.193**	**0.028**
*GroupTaiwan M.:ContrastEarlyFall* vs. *LateFall*	−0.417	0.389	−1.072	0.284
***GroupIndonesian:ContrastLateFall* vs. *EarlyRise***	**−0.917**	**0.458**	**−2.003**	**0.045**
***GroupZhumadian M.:ContrastLateFall* vs. *EarlyRise***	**0.761**	**0.337**	**2.258**	**0.024**
***GroupTaiwan M.:ContrastLateFall* vs. *EarlyRise***	**1.407**	**0.420**	**3.345**	**0.001**
*GroupIndonesian:ContrastLateRise* vs. *RiseFall*	−0.420	0.510	−0.823	0.410
*GroupZhumadian M.:ContrastLateRise* vs. *RiseFall*	0.403	0.334	1.206	0.228
***GroupTaiwan M.:ContrastLateRise* vs. *RiseFall***	**0.948**	**0.405**	**2.342**	**0.019**

Significant results are presented in bold.

**Table 8:** Results of mixed-effects logistic regression analysis with Taiwanese Mandarin and [la] vs. [ta] as baselines.

	*R*^2^ = 0.47				
	B	SE	*z*	*p*
*Intercept*	2.934	0.341	8.604	<0.0001
***GroupZhumadian M.***	**−0.802**	**0.316**	**−2.542**	**0.011**
***GroupSwedish***	**−0.615**	**0.327**	**−1.883**	**0.060**
*GroepIndonesian*	−0.590	0.456	−1.293	0.196
***ContrastEarlyFall* vs. *LateFall***	**−3.926**	**0.358**	**−10.957**	**<0.0001**
*ContrastLateFall* vs. *EarlyRise*	−0.387	0.393	−0.987	0.324
*ContrastLateRise* vs. *RiseFall*	−0.756	0.416	−1.817	0.069
***Sequence***	**−0.421**	**0.044**	**−9.664**	**<0.0001**
***GroupZhumadian M.:ContrastEarlyFall* vs. *LateFall***	**1.155**	**0.370**	**3.119**	**0.002**
*GroupSwedish:ContrastEarlyFall* vs. *LateFall*	0.417	0.389	1.072	0.284
*GroupIndonesian:ContrastEarlyFall* vs. *LateFall*	−0.302	0.498	−0.606	0.544
*GroupZhumadian M.:ContrastLateFall* vs. *EarlyRise*	−0.646	0.411	−1.570	0.116
***GroupSwedish:ContrastLateFall* vs. *EarlyRise***	**−1.407**	**0.420**	**−3.347**	**0.001**
***GroupIndonesian:ContrastLateFall* vs. *EarlyRise***	**−2.324**	**0.518**	**−4.485**	**< 0.0001**
*GroupZhumadian M.:ContrastLateRise* vs. *RiseFall*	−0.545	0.395	−1.380	0.168
***GroupSwedish:ContrastLateRise* vs. *RiseFall***	**−0.948**	**0.404**	**−2.343**	**0.019**
***GroupIndonesian:ContrastLateRise* vs. *RiseFall***	**−1.368**	**0.557**	**−2.455**	**0.014**

Significant results are presented in bold.

The results of the first model show that the Swedish participants (*M* = 0.88) performed comparably at the phoneme contrast baseline with the Indonesian (*M* = 0.86) and Zhumadian Mandarin participants (*M* = 0.87), but marginally underperformed compared to the Taiwan Mandarin participants (*M* = 0.93; *β* = 0.62, *p =* 0.06). Swedish participants performed less well on the tonal contrasts than on the phoneme contrast (EarlyFall vs. LateFall (*M* = 0.25; *β* = −3.51, *p* < 0.0001), LateFall vs. EarlyRise (*M* = 0.61; *β* = −1.79, *p* < 0.0001) and the LateRise vs. RiseFall (*M* = 0.60; *β* = −1.70, *p* < 0.0001). Importantly, the Group–Contrast interactions indicate that the participants of the two tonal languages, Taiwan and Zhumadian Mandarin, outperformed Swedish participants on the LateFall vs. EarlyRise contrast (TM: *M* = 0.90, *β* = 1.41, *p* < 0.001; ZM: *M* = 0.73, *β* = 0.76, *p* = 0.02), while Swedish participants, in turn, outperformed non-tonal Indonesian participants on the same contrast (*M* = 0.44, *β* = −0.92, *p* = 0.05). Additionally, Zhumadian Mandarin participants performed better at the tonal contrast that is specific to their language, EarlyFall vs. LateFall, than the baseline Swedish participants (*M* = 0.36, *β* = 0.74, *p* = 0.03), while the results of the other two groups on this contrast were comparable to those of the Swedish group. Additionally, Taiwan Mandarin participants (*M* = 0.86) performed better on the LateRise vs. RiseFall contrast than the Swedish participants (*M =* 0.60; *β* = 0.95, *p* = 0.02). Finally, and unsurprisingly, 4-word sequences (*M* = 0.69) were responded to with higher accuracy than 5-word sequences (*M* = 0.56; *β* = −0.42, *p* < 0.0001).

The model with Taiwan Mandarin as the baseline shows that the Taiwan Mandarin group outperformed the Zhumadian Mandarin group on the phoneme baseline contrast (*β* = −0.80, *p* = 0.01); the difference with the Swedish group is just shy of significance. The low score for the Indonesian participants is not significantly different from the Taiwan Mandarin group, which is no doubt due to the wider spread of the scores by the Indonesian group compared to the concentration of the Taiwan Mandarin scores around 1 (Figure 7). Similar to the Swedish group, the Taiwan Mandarin group performed less well on the EarlyFall vs. LateFall (*M* = 0.29; *β* = −3.93, *p* < 0.0001) and the LateRise vs. RiseFall (*M* = 0.86; *β* = −0.76, *p* = 0.07) contrasts than on the phoneme contrast. Their performance on the LateFall vs. EarlyRise contrast, however, was as good as that on the phoneme contrast (*M* = 0.90; *β* = −0.39, *p* = 0.32). While the Taiwan Mandarin group still outperformed the non-tonal Indonesian and “semi-tonal” Swedish groups on the LateFall vs. EarlyRise and LateRise vs. RiseFall contrasts (Indonesian: *β* = −2.32, *p* < 0.0001; Swedish: *β* = −1.41, *p* < 0.001), the Zhumadian Mandarin group stood out on the Zhumadian-specific contrast, EarlyFall vs. LateFall (*M* = 0.36; *β* = 1.16, *p* = 0.002), the only contrast for which the Taiwan Mandarin group scored below Zhumadian Mandarin (see also Figure 7).

## Discussion

There are three main results of our experiment on the sequence recall of pitch shapes with Indonesian, Swedish, and Mandarin participants.

1. Accuracy scores were positively influenced by (i) similarities between experimental pitch contrasts and phonological contrasts in the languages, (ii) the phonetic salience of the experimental pitch contrast, and (iii) the participant’s aptitude for the experimental task as measured by the score on the phoneme contrast.
2. On one contrast, LateFall vs. EarlyRise, the Swedish group distinguished themselves as intermediate by outperforming the Indonesian group and being outperformed by the two Mandarin groups, with the two Mandarin groups not differing among themselves.
3. On none of the three-pitch contrasts did semi-tonal Swedish participants and the two tonal Mandarin groups outperform the non-tonal Indonesian group without differing among themselves.

We discuss these three findings in this order below.

### Dependence of Tone Contrast Sequence Recall Accuracy Scores on Other Factors

Without a doubt, the linguistic effects of our first finding will show up in similar experiments performed with different selections of languages. Given the small size of our experiment, we cannot be confident that the effect sizes will be preserved proportionally in experiments with different sets of pitch contrasts and languages, but our results do show that a tonal SRT will need to address the effects of linguistic properties to a larger extent than a stress-based SRT (*cf*. Best, 2019). Despite the cross-linguistic variation in the distribution of stressed syllables within words outlined in Peperkamp and Dupoux (2002), the cross-linguistic variation in tone systems is larger than that of stress.

The effect of the general ability of participants to perform an SRT, as measured by the accuracy scores of the phoneme contrast (aptitude), turned up among four groups of participants with similar age ranges and levels of education. This suggests that for older participants, this task may be more challenging and hence likely to produce lower accuracy scores compared to our participants. Less demanding versions of this experimental task may therefore need to be explored with older participants. As far as we are aware, this is the first time that an SRT aptitude effect has shown up. Rahmani et al. (2015) ignored the phoneme contrast for not being significantly different between language groups. In Peperkamp et al. (2010), the dependent variable was the difference between the accuracy scores for the phoneme contrast and the stress contrast, on the assumption that this effect will exist in absolute terms, while excluding participants showing poor performance from the analysis, resulting in a significant data loss. By including the phoneme contrast scores as a variable in our multiple regression analyses and the model analyses, we were able to retain all participants in the experiment so as to closely model their performance. Various components of aptitude have been addressed in more recent studies as a variable that could potentially modulate tone perception, as in Bowles et al. (2016) and Qin et al., (2021).

The effect of the existence of an experimental pitch contrast in a language’s phonology (havecontrast) is apparent from the interactions between the pitch contrasts and the language groups in the mixed-effects models. The Zhumadian group, whose language is the only one to have a temporal alignment contrast for falls, outperformed both the Swedish and Taiwan Mandarin groups on the EarlyFall vs. LateFall contrast, in addition to the low-scoring Indonesian group. The three non-Zhumadian groups did not differ significantly from each other, as shown by the lack of any interaction between Indonesian and the EarlyFall vs. LateFall contrast in either analysis (Tables 7 and 8). The effect of contrast salience (salience) was most clearly in evidence in the overall lower scores for the EarlyFall vs. LateFall contrast compared to the other two pitch contrasts.

### Three Typological Groups?

Our second finding was that both Mandarin groups outperformed the Indonesian and Swedish groups on the LateFall vs. EarlyRise contrast, with the Indonesian group scoring below the Swedish group. If we interpret the contrast between rising and falling pitch to be prototypical, the pattern Indonesian < Swedish < Zhumadian and Taiwan Mandarin suggests a three-way distinction between atonal, semi-tonal, and tonal languages. If this result were to be replicated with other mixes of languages, it would imply that a binary diagnostic is unlikely to emerge from a tone-based SRT with a broad typological mix of languages. In turn, this might put experiments with small numbers of languages that have yielded significant results between tonal and non-tonal languages in a different perspective, in the sense that they may represent values on a tone/non-tone continuum rather than as values of a binary variable.

### Testing Varieties of the Same Language

Turning the above conclusion around so as to adopt a positive perspective, we might expect tonal and non-tonal varieties of the same language that otherwise have few differences between them to be consistently distinguishable with the help of a tonal SRT. Such languages include Japanese, Korean, Swedish/Norwegian, Franconian varieties of Dutch and German, and Serbian/Croatian (van der Hulst et al., 2011; Gussenhoven and Chen, 2020). Importantly, it is in such cases that the tonal nature of languages has been debated, most notably with respect to two properties, one distributional and the other representational. The first is exemplified by Tokyo Japanese and Northern Bizkayan Basque, which have been characterized as “pitch accent languages,” a distinct type by the side of tonal and non-tonal languages. Dominant characterizations of this group indicate the restriction of contrastive tone in a single location of the word or word-like domain. Hyman (2006, 2009) has signaled the absence of a clear definition, in particular that of the demarcation line with tone languages proper. Thus, the single location could be “fixed,” like the penultimate syllable of Lekeitio Basque, be restricted to the non-final stressed syllable, as in Swedish, or to one of two syllables at a word edge, as in Kagoshima Japanese and Barasana, or be lexically specified, as in Tokyo Japanese (Elordieta, 1998; Gomez-Imbert and Kenstowicz, 2000; Hualde, 2012; Jun and Kubozono, 2020). Also, there may be two locations for a tone contrast, one at the beginning and one toward the end, as in Osaka and Ibukujima Japanese (Pierrehumbert and Beckman, 1988; Uwano, 1999), while the contrastive tone could be privative, as in the above varieties of Japanese, or represent a contrast between two tone melodies, as in Barasana (*cf*. Hualde, 2012). The other controversy concerns the issue whether surface tone contrasts in varieties of Swedish/Norwegian and Franconian are due to underlying tones (e.g., Bruce, 1977; Riad, 2014; Gussenhoven and Peters, 2019) or to differences in underlying foot structure which generate the different surface tone structures (e.g., Köhnlein, 2011, 2016, 2017; Hermans, 2012; Morén-Duolljá, 2013; Kehrein, 2018). Future explorations of our tone-based SRT might therefore fruitfully compare non-tonal and putatively tonal varieties of the same language.

## Data Availability Statement

The datasets presented in this study can be found in online repositories. The names of the repository/repositories and accession number(s) can be found at: https://osf.io/uxv4j/?view_only=86c8981b6c9c46c38fe2c2900afd4bcc.

## Ethics Statement

The studies involving human participants were reviewed and approved by Research Ethics Committee for Human Subject Protection of National Yang Ming Chiao Tung University. The participants provided their written informed consent to participate in this study.

## Author Contributions

CG: conceptualization, methodology, data curation, supervision, and writing—original draft. Y-AL: data curation, formal analysis, funding acquisition, project administration, resources, visualizations, software, Taiwan Mandarin and Indonesian experiments, and writing—review and editing. S-IL-K: methodology, formal analysis, and writing—review and editing. CL: resources, Zhumadian Mandarin experiment, and writing—review and editing. HR: resources. TR: writing—review and editing. HZ: Swedish experiment and writing—review and editing. All authors contributed to the article and approved the submitted version.

## Funding

The work by CG was supported by MOST-208-2811-H-009-500 (Ministry of Science and Technology, Taiwan) awarded to Ho-hsien Pan. Taiwan Mandarin and Indonesian participants were run using MOST 109-2410-H-009-048 granted to Y-AL.

## Conflict of Interest

The authors declare that the research was conducted in the absence of any commercial or financial relationships that could be construed as a potential conflict of interest.

## Publisher’s Note

All claims expressed in this article are solely those of the authors and do not necessarily represent those of their affiliated organizations, or those of the publisher, the editors and the reviewers. Any product that may be evaluated in this article, or claim that may be made by its manufacturer, is not guaranteed or endorsed by the publisher.

## Footnotes

^1^In a methodologically comparable experiment with 40 participants, no effect of order of presentation within a pair was found (Fournier and Gussenhoven, 2010). In that experiment, the scores of all participants correlated with the mean scores over all judges, with a range of 0.56–0.88.
